# Integrated View of Baseline Protein Expression in Human Tissues Using Public Data Independent Acquisition Data Sets

**DOI:** 10.1021/acs.jproteome.4c00788

**Published:** 2025-01-07

**Authors:** Ananth Prakash, Andrew Collins, Liora Vilmovsky, Silvie Fexova, Andrew R. Jones, Juan Antonio Vizcaino

**Affiliations:** † European Molecular Biology Laboratory-European Bioinformatics Institute (EMBL-EBI), Wellcome Genome Campus, Hinxton, Cambridge CB10 1SD, U.K.; ‡ Institute of Systems, Molecular and Integrative Biology, University of Liverpool, Liverpool L69 7ZB, U.K.

**Keywords:** data independent acquisition, mass spectrometry, proteomics, data reanalysis, baseline expression, Expression Atlas, PRIDE

## Abstract

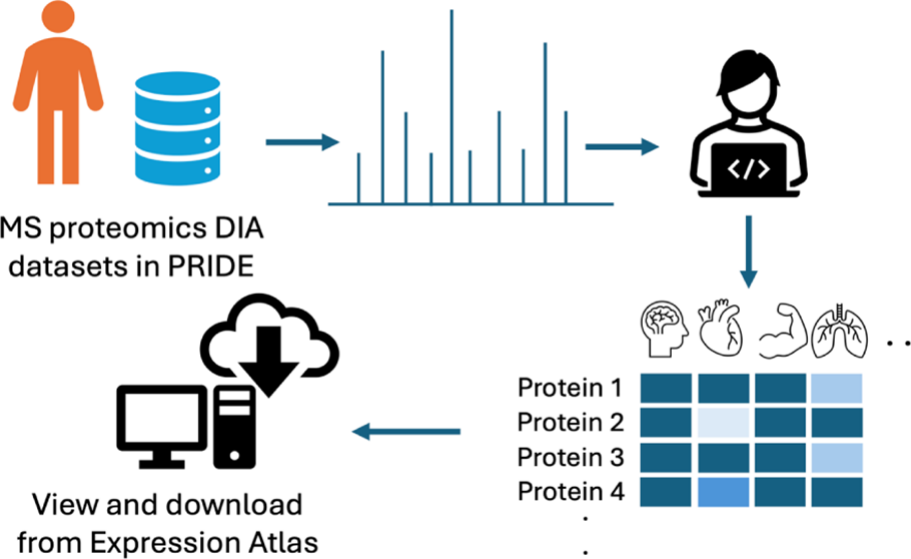

The PRIDE database is the largest public data repository of mass spectrometry-based proteomics data and currently stores more than 40,000 data sets covering a wide range of organisms, experimental techniques, and biological conditions. During the past few years, PRIDE has seen a significant increase in the amount of submitted data-independent acquisition (DIA) proteomics data sets. This provides an excellent opportunity for large-scale data reanalysis and reuse. We have reanalyzed 15 public label-free DIA data sets across various healthy human tissues to provide a state-of-the-art view of the human proteome in baseline conditions (without any perturbations). We computed baseline protein abundances and compared them across various tissues, samples, and data sets. Our second aim was to compare protein abundances obtained here from the results of previous analyses using human baseline data-dependent acquisition (DDA) data sets. We observed a good correlation across some tissues, especially in the liver and colon, but weak correlations were found in others, such as the lung and pancreas. The reanalyzed results including protein abundance values and curated metadata are made available to view and download from the resource Expression Atlas.

## Introduction

The most popular quantitative approach for mass spectrometry (MS)-based proteomics has historically been data-dependent acquisition (DDA) bottom-up proteomics. However, data independent acquisition (DIA) approaches^1^ have matured enormously, thanks to multiple technical developments in, e.g., sample preparation and improvements in instrumentation and computational data analysis approaches.^2^ In contrast to DDA, where only the most intense peptide ions are measured, in DIA, fragmentation products are generated from every peptide ion that is sampled in the MS^1^ scans. As a result, a DIA analysis approach can potentially provide a more comprehensive quantitative view on the proteome when compared with DDA approaches.

There are two main approaches to DIA proteomics data analysis: spectrum-centric and peptide-centric. Spectrum-centric methods follow the DDA general approach by generating “pseudo-MS/MS spectra,” where fragment ions are associated with the precursor ion from which they are most likely derived. The pseudo spectra can be analyzed like DDA data, for instance, through typical sequence database searching tools. Peptide-centric methods are currently much more widely used and rely on deciding in advance which peptidoforms may be present in the sample by using a spectral library.^2^ There are three main approaches for generating spectral libraries that can be used for DIA analysis. First, it is possible to perform a deeply fractionated DDA analysis using the same instrumentation as will be used by the DIA experiments, to generate an *experimentally matched* spectral library. Second, there are some publicly available libraries, created from a given type of sample, for instance, a “pan-species” spectral library assembled from multiple MS runs.^3^ The third approach consists of the use of *in silico* generated spectral libraries, generated using artificial intelligence (AI)-trained models, having learnt retention times and peptide intensities from past DDA data sets.^2^

In parallel to other developments, for the past decade or so, the proteomics community has learned to follow and implement open data practices. The PRIDE database,^4^ one of the founding members of the ProteomeXchange consortium,^5^ is the most popular proteomics data repository worldwide. The availability of extensive public proteomics data sets in PRIDE and other ProteomeXchange resources has triggered multiple applications, such as meta-analysis studies including some studies applying different AI approaches.^6,7^ Additionally, by systematically reanalyzing public data sets, original findings can be updated, confirmed, and/or strengthened.^8^ Moreover, novel insights beyond the scope of the original studies can be obtained through alternative reanalysis strategies, for instance, in the case of proteogenomics approaches.^9^

In this context, we are facilitating comparison of transcriptomics and proteomics data in the resource Expression Atlas,^10^ so that protein expression abundance information is made accessible in the long term to life scientists, including those who are not experts in proteomics. We have already performed combined analyses of baseline (i.e., without any perturbation) protein expression studies of public DDA experiments coming from human,^11^ mouse, rat,^12^ and pig tissues,^13^ as model organisms. Additionally, we have also performed combined analyses of DDA experiments generated from cell lines and tumor tissue,^14^ and more recently, a study focused on colorectal cancer-related data sets as an approach for biomarker discovery.^15^ In all cases, protein abundance results have been made available through the Expression Atlas. There, protein abundance data can be visualized together with gene expression information, although rarely from the same samples.

However, in parallel with the trends in the field, an increasing fraction of data deposited in PRIDE and in other ProteomeXchange resources comes from the DIA approaches. In this context, a few years back, we carried out a pilot study including ten DIA human data sets including mostly cell lines (all of them, SWATH-MS experiments from SCIEX instruments),^16^ using an analysis approach based on the use of the “pan-human” spectral library.^17^

Here, we report the reanalysis and integration of 15 public label-free DIA human baseline tissue data sets including 178 healthy/normal control samples. The objective of this study is 2-fold: on one hand, we aim to facilitate access to protein abundance data from baseline human tissues coming from state-of-the-art DIA approaches. On the other hand, we want to study their similarity with analogous protein abundance data generated using DDA approaches. Unlike our previous study involving DIA data sets,^16^ we used *in silico*-generated spectral libraries for the analysis. The protein abundance results, as in our previous studies, have been incorporated into the Expression Atlas. Additionally, we made a comparison of the expression values coming from DIA data sets and the protein abundance results from previous DDA studies including human baseline tissues,^18,19^ for instance, using ProteomicsDB data and also our previous combined analysis of DDA public human data sets.^11^

## Methods

### Data Sets

Public MS-based human DIA proteomics data sets were queried from the PRIDE database in January 2023. Out of the 288 data sets that were available, several data sets were selected for downstream reanalysis. The selection criteria included (i) nonenriched samples, only coming from tissues (not including cell lines or fluid samples such as blood/plasma, cerebrospinal fluid, etc.); (ii) data sets from SCIEX (TripleTOF series) and Thermo Fisher Scientific (Q Exactive series and Orbitrap) instruments; (iii) availability of experimental metadata to link samples to their respective data files; and (iv) in the case of data sets from SCIEX instruments, only data sets that had both .wiff and .scan files deposited were considered (this is a requirement that was added for all SCIEX data submissions to PRIDE in 2021, but there are some previously submitted data sets that do not comply with this requirement). Where enough experimental metadata was not made available in the initial data submissions and/or in the corresponding publications, we contacted their respective authors to obtain this information. At the end, we selected 15 data sets for reanalysis (Table 1).

**Table 1:** Summary of Protein Identifications among Data sets

Expression Atlas accession number	PRIDE proteomics data set identifier	Tissue	Mass spectrometer	Fractionation	No. of MS runs^a^	Number of samples^a^	Number of PSMs^a^	Number of unique peptides^b^	Number of unique genes (canonical proteins)^c^
E-PROT-137	PXD031419^29^	Brain	TripleTOF 5600	No	75	19	1,424,053	49,709	5069
E-PROT-138	PXD012254^30^	Colon	Q Exactive	No	19	19	871,632	53,273	5567
E-PROT-139	PXD001506^31^	Duodenum	TripleTOF 5600	No	6	6	104,435	14,424	1955
E-PROT-140	PXD001764^32^	Esophagus	TripleTOF 5600	No	3	3	58,131	15,545	2546
E-PROT-141	PXD019594^33^	Heart	TripleTOF 5600	No	10	10	93,177	10,456	1131
E-PROT-142	PXD025705^34^	Liver	Q Exactive Plus	No	33	11	3,099,160	85,833	6403
E-PROT-143	PXD004684^35^	Lung	Q Exactive	No	8	4	155,588	25,179	2905
E-PROT-144	PXD032076^36^	Pancreas	Orbitrap Fusion	No	25	25	417,648	27,139	3507
E-PROT-145	PXD025431^37^	Skin	Q Exactive HF	No	10	10	437,823	48,343	4134
E-PROT-146	PXD002732^38^	Thyroid	TripleTOF 5600	No	8	8	160,326	21,965	2999
E-PROT-147	PXD022872^29^	Brain	TripleTOF 5600	No	71	11	1,432,467	50,605	5119
E-PROT-148	PXD033060^39^	Brain	TripleTOF 5600	Yes	17	17	621,054	44,306	4987
E-PROT-149	PXD018830^40^	Breast epithelium	Q Exactive	No	4	4	113,729	27,855	4159
E-PROT-150	PXD039665^41^	Skin	Orbitrap Exploris 480	No	13	13	124,942	13,463	1759
E-PROT-151	PXD034908^42^	Skeletal muscle	TripleTOF 6600	No	54	18	1,174,230	18,406	1413
Total	**15 data sets**	**12 tissues**			**356 MS runs**	**178 Samples**			

a Healthy/normal control samples only.

b Postprocessed results from control samples, wherein each peptide has at least 2 peptides mapping to it.

c Postprocessed results from control samples, where a gene has at least two unique peptide mappings. Protein expression data in the Expression Atlas can be accessed using the link https://www.ebi.ac.uk/gxa/experiments/E-PROT-XXX/Results, where XXX should be replaced with an E-PROT identifier.

Metadata was annotated using Annotare^20^ and saved as investigation description format (IDF) and sample and data relationship format (SDRF) files. IDF comprises descriptions about data set experiment protocol, contact information on investigators, and publication details, while SDRF contains information on samples including case/control, donor age, gender, treatment conditions, experimental factors, etc. Both IDF and SDRF files were integrated into the Expression Atlas.

### Proteomics Raw Data Processing

To process spectral data on a Linux (Rocky Linux 8) platform, the vendor raw files were first converted to an mzML format using conversion tools. For Thermo Fisher Scientific instruments (.raw), ThermoRawFileParser^21^ 1.4.2 was used with default settings. For SCIEX instruments (.wiff and .scan), ProteoWizard’s msConvert^22^ conversion tool was used with the “peakPicking vendor” option enabled.

As a target database, the UniProt human “one protein sequence per gene” database (UP000005640, May 2023) with 20,838 sequences was used to which cRAP contaminant sequences^23^ (245 sequences) were added. The target database was used in generating an *in silico* spectral library using DIA-NN^24^ (version 1.8.1) with default parameters. We also used an entrapment database,^25,26^ wherein *Arabidopsis thaliana* UniProt “one protein sequence per gene” database (UP000006548, December 2022) with 41,621 sequences, which was added to the target human protein search database. Similarly, an *in silico* spectral library of the entrapment database was generated with default parameters using DIA-NN.

To improve runtime performance, we designed our analysis by separating a conventional DIA-NN^24^ run into two phases so that it could be reanalyzed efficiently on a Linux high-performance computing (HPC) platform, using the Slurm job scheduler.^27^ Prior to performing the identification for any given data set, a calibration run was first performed to identify the search tolerances. By default, DIA-NN will autodetect the tolerances using the first sample in the batch. This leads to issues when running DIA-NN in parallel, as each sample will be run with different tolerance settings. To solve this, we ran DIA-NN using default autodetect settings on a single sample against the target database. The output of this calibration run provided the MS1, MS2, and scan window values that would be used for the full run.

Using the tolerance values identified above, we then employed a multinode approach to process all raw files in parallel across the HPC cluster. This parallel process allowed us to reduce the runtime of DIA-NN’s first phase to the runtime of a single file, a speed-up factor directly proportional to the number of files in each data set. In this first phase, each MS run was searched against UniProt human with an *Arabidopsis thaliana* entrapment database and the *in silico* spectral library mentioned above. During this phase, cross-run normalization and MaxLFQ-based protein quantification were disabled (--no-norm, --no-maxlfq), match between runs was not performed, and the main report was not generated (--no-main-report). The *Q*-value was set to 0.01, peptide cleavage sites were assigned to trypsin, and the rest of the parameters were set to default. At the completion of the first phase, protein quantification (.quant files) from individual runs were saved in a temporary directory. During the second phase, the existing .quant files generated from the first phase (--use-quant) were collated, and a cross-run analysis was performed on a single node, wherein cross-run normalization, match-between runs, and MaxLFQ-based protein quantification were performed, and main report files, including protein groups, gene groups, unique genes, and precursors matrices, were generated.

In a conventional DIA-NN run, each input file is sequentially analyzed against the spectral library for protein identification and quantification. By leveraging multinode processing in the first phase, wherein each file was simultaneously analyzed and then collating the .quant files, this significantly reduced the total runtime by approximately 50% (Figure S1).

### Postprocessing

The report .tsv output file from DIA-NN was used as the basis for postprocessing. Contaminants were removed along with mappings to more than one protein or gene identifier. In each MS run, we removed entries with fewer than two unique peptide sequences per protein, and the abundances of proteins were aggregated using their median values within each MS run in a data set. DIA-NN outputs abundances as label-free quantification (LFQ) values. We converted LFQ to intensity-based absolute quantification (iBAQ) values by normalizing the LFQ abundances with the theoretical number of tryptic peptides of each canonical protein. As explained in previous publications,^11−13^ we represent protein abundances as abundances of their respective parent gene names, which we will use as equivalent to “canonical proteins” as described in UniProt (https://www.uniprot.org/help/canonical_and_isoforms). For ease of comparison of protein abundances across tissues and data sets with potentially large batch effects between different experiments, iBAQ protein abundances were converted into ranked bins.^11^ Briefly, the iBAQ abundances were numerically sorted and grouped into five bins of equal size. Proteins in bin 1 were of lowest abundance, and those in bin 5 were highly abundant. The heatmap of samples was generated using binned abundance values. Postprocessing was performed using R scripts.

### Integration into Expression Atlas

Postprocessed results comprising protein abundances, expressed as their canonical gene identifiers (proteins were mapped to gene identifiers by DIA-NN), were integrated into Expression Atlas^28^ along with metadata files (IDF and SDRF) and a quality assessment summary. Expression Atlas data set identifiers (E-PROT) for each data set are shown in Table 1.

### Comparison of Baseline Protein Abundances Generated Using DDA Data

Ensembl gene identifiers of the respective canonical proteins were used for comparing proteins identified between this study and previous DDA studies performed in human baseline tissues. Ensembl gene identifiers and normalized iBAQ protein abundance values in SupplementaryTable 2 available from ref (11) were gathered for tissues that are common between the two studies for comparison purposes. The iBAQ protein abundances of DIA data sets were then compared with the fraction of total normalized iBAQ protein abundances from DDA data sets.

Additionally, normalized protein intensities from ProteomicsDB^18^ were queried for tissues that were common in our study (8 tissues). Values were obtained using the ProteomicsDB Application Programming Interface (uploaded in April 2022). For different tissue samples, we aggregated the normalized intensities using the median of their respective tissues. The intensities were log2 normalized and compared.

Furthermore, protein abundances calculated across various baseline human tissues using the TMT-labeling method were obtained from ref (19) (Supplementary file “NIHMS1624446-supplement-2”, sheet: “C protein normalized abundance”). Protein abundances of the respective tissues measured across different TMT channels and MS runs were aggregated using the median and log2 transformed. Different tissue samples from the esophagus, heart, brain, and colon were aggregated into their respective tissues. Binned protein abundances of the samples were used for calculating Pearson correlation values within each tissue. For pairwise comparison of samples between different data sets, only the abundances (binned values) of proteins commonly identified between two samples were considered. The brain and skin were the only tissues in this study, which are available in multiple data sets. The correlation of protein abundances between brain and skin samples from multiple data sets was calculated, and median correlation values are presented. Correlations were calculated by using the R programming language.

### Comparison of Missing Values between DDA and DIA Data Sets

To compare the proportion of missing protein abundance values between DDA and DIA techniques, we used the protein abundances from human DDA samples calculated in our previous study.^11^ Protein abundances from DDA samples were represented as their canonical gene identifiers, as described for DIA samples above. To compare the completeness of protein detection among samples of their respective tissues within a data set, first, for a given tissue, we calculated the total number of missing abundances (NA) among its samples across all canonical proteins and then normalized it by the total number of observations, as shown in the following equation:
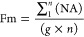
where “Fm” is the fraction of missingness, “*n*” is the total number of samples of a particular tissue within a data set, “*g*” is the total number of genes identified in those respective tissue samples within the data set, and “NA” is the missing abundance of a canonical protein. Where a data set had more than one tissue, the “Fm” was calculated individually for each group of tissue samples, and then a median of “Fm” over all tissues was calculated for the data set.

### False Discovery Rate Analysis Using Entrapment Database

All data sets were analyzed individually. To estimate the protein false discovery rate (FDR) across data sets, we used an *Arabidopsis thaliana* entrapment search database as explained above. From the protein group matrix report output file from DIA-NN, we treated the protein groups that had gene identifiers belonging exclusively to *Arabidopsis thaliana* as decoys, and the rest of the protein groups were regarded as targets. The distribution of the number of data sets in which all decoys and targets were found in common was computed, and the FDR for a target protein identified across a number of data sets was calculated using the equation
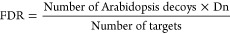
where “Dn” = 0.5 is the database normalizing factor, i.e., the size of the UniProt human one protein per gene set database (20,838 sequences) normalized per the size of the UniProt *Arabidopsis thaliana* one protein per gene set database (41,621 sequences).

## Results

### DIA Human Proteomics Data Set Reanalysis

**Figure 1:**
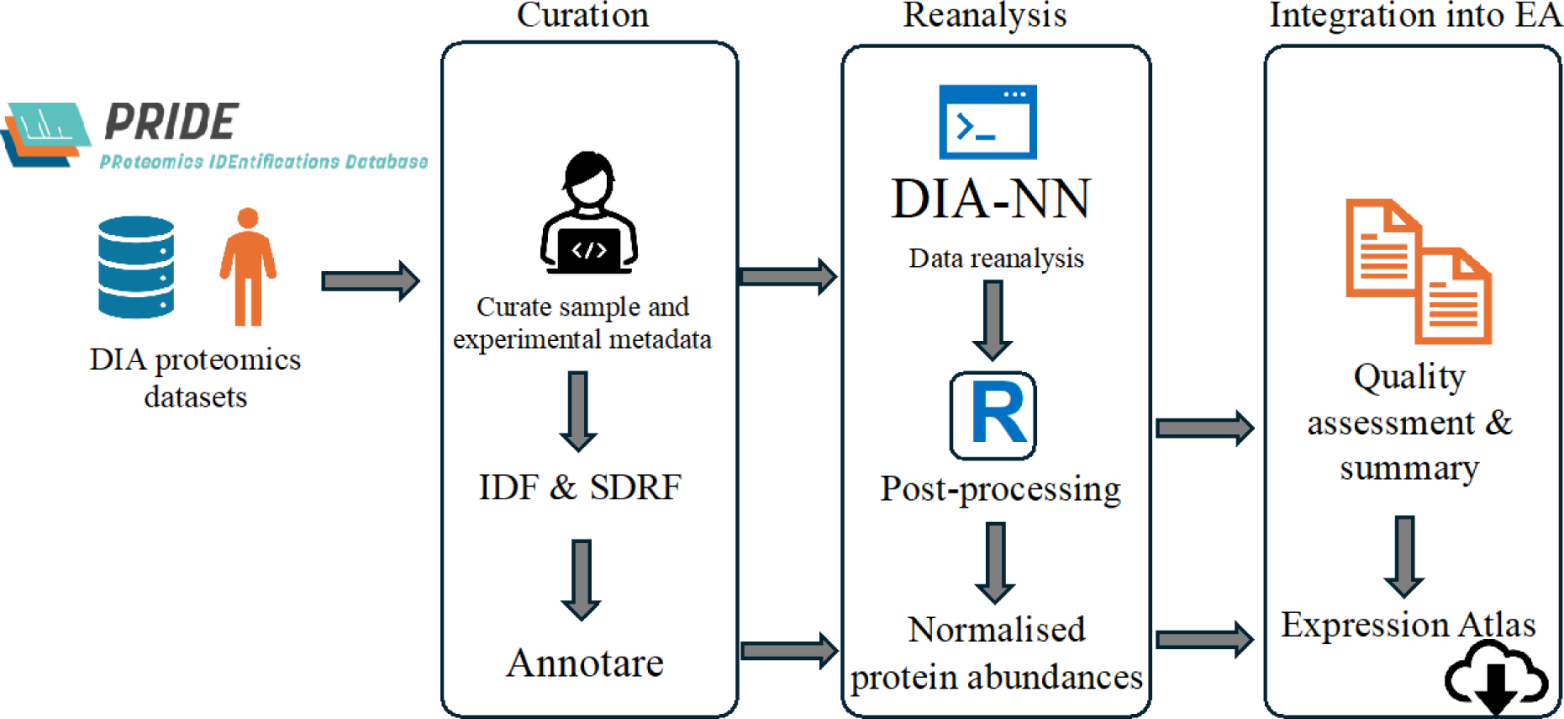
Overview of DIA data sets’ reanalysis pipeline. EA = Expression Atlas.

We selected 15 DIA proteomics data sets, which represented the available range in the public domain for human tissues in healthy/baseline conditions. The spectra from these data sets were acquired either using SCIEX or Thermo Fisher Scientific instruments, as explained in “Methods”. In total, there were 1,361 MS runs from all samples in aggregated data sets, which included 356 MS runs from 178 healthy or normal control samples. Each data set was analyzed individually and postprocessed. The complete list of data sets along with the number of peptides and proteins identified and quantified is shown in Table 1. Protein abundances computed only from healthy or normal tissue samples are discussed in this manuscript. However, abundances from all samples along with a quality assessment summary and sample metadata are made available in Expression Atlas (https://www.ebi.ac.uk/gxa/) to view and download. The overall data reanalysis protocol is summarized in Figure 1.

### Protein Coverage across Samples

**Figure 2:**
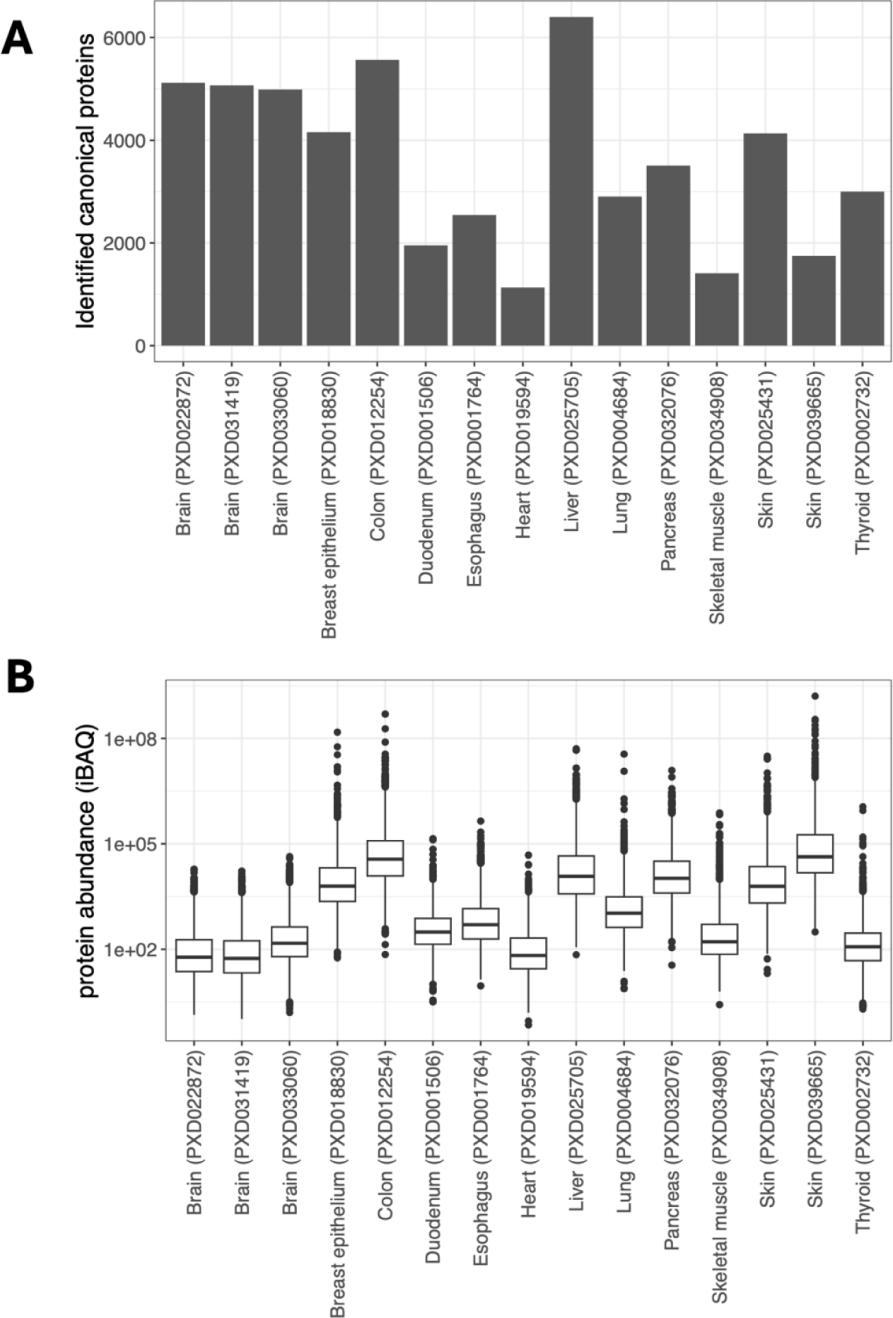
Distribution of protein identification and abundances across tissues and data sets. (A) Number of canonical proteins identified across different tissues and data sets. (B) iBAQ protein abundances of canonical proteins across different tissues and data sets.

From each sample analyzed in a data set, we kept those proteins, which were identified by at least two peptides. Those mapping to more than one gene identifier were also filtered out for downstream analysis. We identified a total of 9,299 canonical proteins, of which 521 proteins were observed in all 12 tissues, and 2,679 were only found in one tissue (Table S1). We identified the largest number of proteins in the liver (6,401 proteins, 68.8%) followed by the brain (6,018, 64.7%, aggregated over three data sets) and colon (5,564, 59.8%). The fewest proteins were identified in the heart (1,131, 12.2%), skeletal muscle (1,412, 15.2%), and duodenum (1,955, 21.0%) (Figure 2A). As seen in Table 1, each tissue is represented by one data set, except the brain, which is represented by 3 data sets: PXD022872 (5,119, 55.0%), PXD031419 (5,069, 54.5%), and PXD033060 (4,987, 53.6%), and skin, which is represented by 2 data sets: PXD025431 (4132, 44.4%) and PXD039665 (1,748, 18.8%). We observed significant variations in the protein abundances across each tissue. Figure 2B shows the aggregated (median) protein abundances of each tissue over several samples. Among data sets, PXD025705 (6,401, 68.8%) from the liver had the largest number of proteins identified, followed by PXD012254 (5,564, 59.8%) from the colon. The protein abundances in each data set vary, reflecting those observed for every tissue.

### Protein Abundance Comparison across Tissues

To compare protein abundances across different tissues, we first transformed the LFQ protein abundance values obtained from DIA-NN to iBAQ values, as explained in the “Methods” section. To make easier the comparison across data sets and tissues, we grouped iBAQ values equally into five categorical bins. Proteins within bin 1 are of lowest abundances, and those in bin 5 are of highest abundances. These binned abundances are available in Table S2.

**Figure 3:**
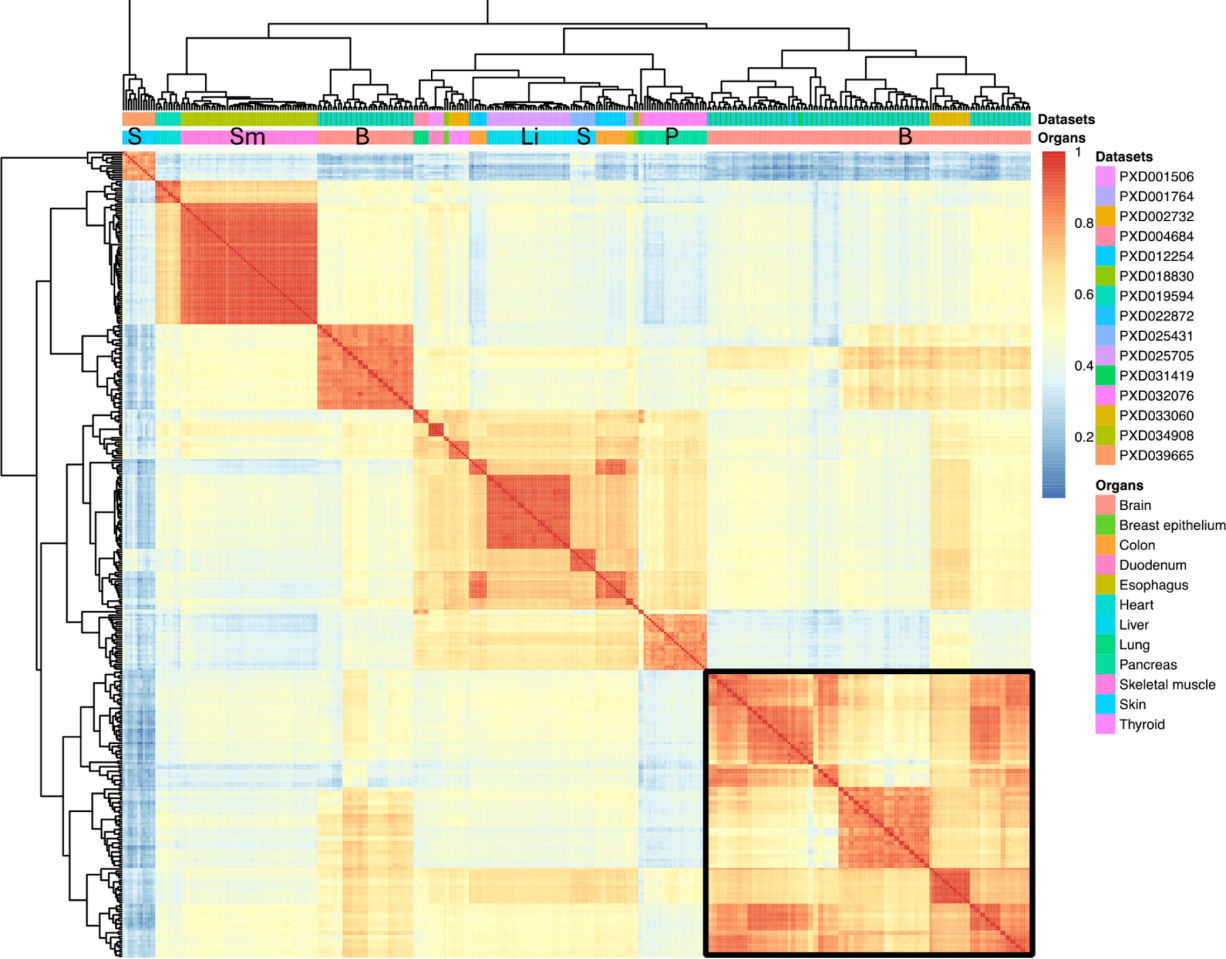
Heatmap of binned protein abundances across all samples between various tissues and data sets. Brain samples clustered together are highlighted using a black border. S: skin, Sm: skeletal muscle, B: brain, Li: liver, and P: pancreas.

We carried out pairwise comparisons across all samples (*n* = 178) using the binned protein abundance values (Figure 3). We observed moderate correlation of protein abundances in brain (median *R*^2^ = 0.40) and low correlation of protein abundances in skin samples (median *R*^2^ = 0.20). Due to the large number of brain samples included in the aggregated data set (*n* = 47),there were more data points for computing Pearson correlation values when compared to pairwise sample comparisons made between different tissues. In comparison, the correlation of protein abundances in DDA brain samples from our previous study^11^ was slightly higher (median *R*^2^ = 0.61). It is important to note that the number of DDA brain samples was then much larger (*n* = 339) than those analyzed here by DIA approaches (*n* = 47).

### *Arabidopsis thaliana* Entrapment Analysis

**Figure 4:**
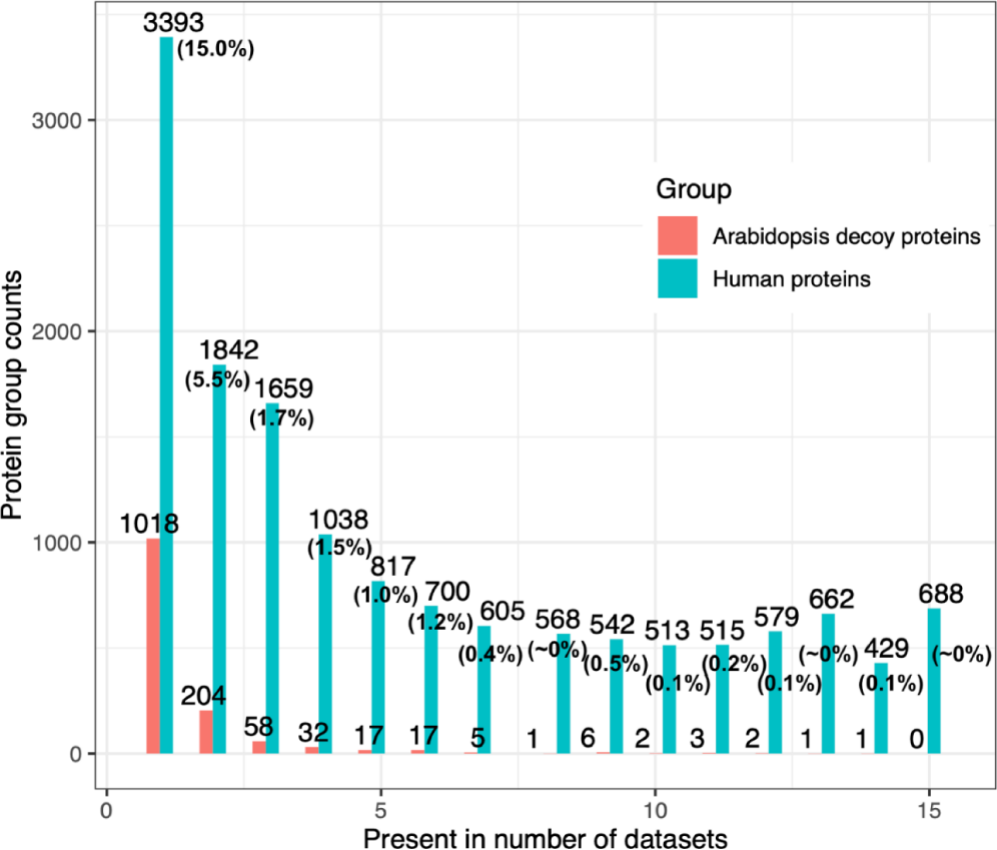
(A) Distribution of decoy and target protein groups present across all data sets identified using the *Arabidopsis thaliana* entrapment search database. The protein FDR values of the target proteins present in common across different numbers of data sets are shown in parentheses. The calculation of the FDR is described in the “Methods” section.

We designed this work to reanalyze each data set individually, as in previous studies. The protein FDR was set to 1% with the Benjamini–Hochberg correction for multiple hypothesis testing within each data set. Although this is a stringent filter, if protein observations from multiple data sets are collated at the end, this could result in a protein FDR much greater than 1%. To check this, we used an entrapment target sequence approach, wherein *Arabidopsis thaliana* protein sequences were added to the target human sequence database. The results from the reanalyses of data sets were searched against the entrapment database for protein groups comprised exclusively of canonical *Arabidopsis thaliana* proteins (false targets). We found a total of 1,367 decoy protein groups across all 15 data sets including 1,018 decoy protein groups, which were observed in only one data set (Figure 4). The largest fraction of decoy hits was found in data set PXD039665 (4.3%) and the lowest in data set PXD012254 (1.1%). From the number of decoys detected in common across all data sets, we estimated a resulting combined protein FDR of less than 1% when proteins were observed in at least 7 different data sets and an FDR of less than 5% when proteins were observed in at least 3 data sets. In all cases, *Arabidopsis thaliana* proteins with evidence of at least 2 peptides were considered. When using the aggregated results from all data sets, column C (“Present_in_number_of_data sets”) in Tables S1 and S2 should be used to filter canonical proteins observed in a different number of data sets for obtaining the appropriate values of the “combined protein FDR”. We concluded that the protein FDR values were comparable to our previous human DDA study and appropriate for the objectives of this work. We recommend that DIA analyses use an entrapment approach such as this, to ensure that there is robust control of the FDR, following any postprocessing or merging steps that may be done.

### Protein Abundance Comparison between the DIA Data Sets and Previous DDA Studies

We had previously reanalyzed 24 public proteomics data set representing 31 healthy human tissues^11^ from DDA studies. We first compared the number of canonical proteins identified between data sets analyzed using DDA and DIA (see “Methods”). We had identified a total of 13,071 canonical proteins from DDA studies and 9,299 canonical proteins from the reanalysis of the DIA data sets in this study, all in baseline conditions. When comparing DDA and DIA results, we found 8,449 (64.6%) common canonical proteins obtained from the two groups of data sets, with 4,621 (35.3%) canonical proteins identified only in DDA data sets and 853 (9.1%) canonical proteins identified only in DIA data sets (Table S3 and Figure S2). It is important to note here that in comparison to the DIA data sets, there were many more MS runs and samples analyzed in the DDA data sets, and additionally, many of the DDA data sets were fractionated, increasing the depth and coverage of the study. By analyzing the missingness of protein abundance values among samples and comparing it with samples in DDA data sets from our earlier study,^11^ we observed similar trends in the fraction of missing values among samples analyzed by DIA and DDA techniques, implying that DIA does not confer substantial advantages in terms of avoiding missing values, at least in the data sets we analyzed (Figure S3).

**Figure 5:**
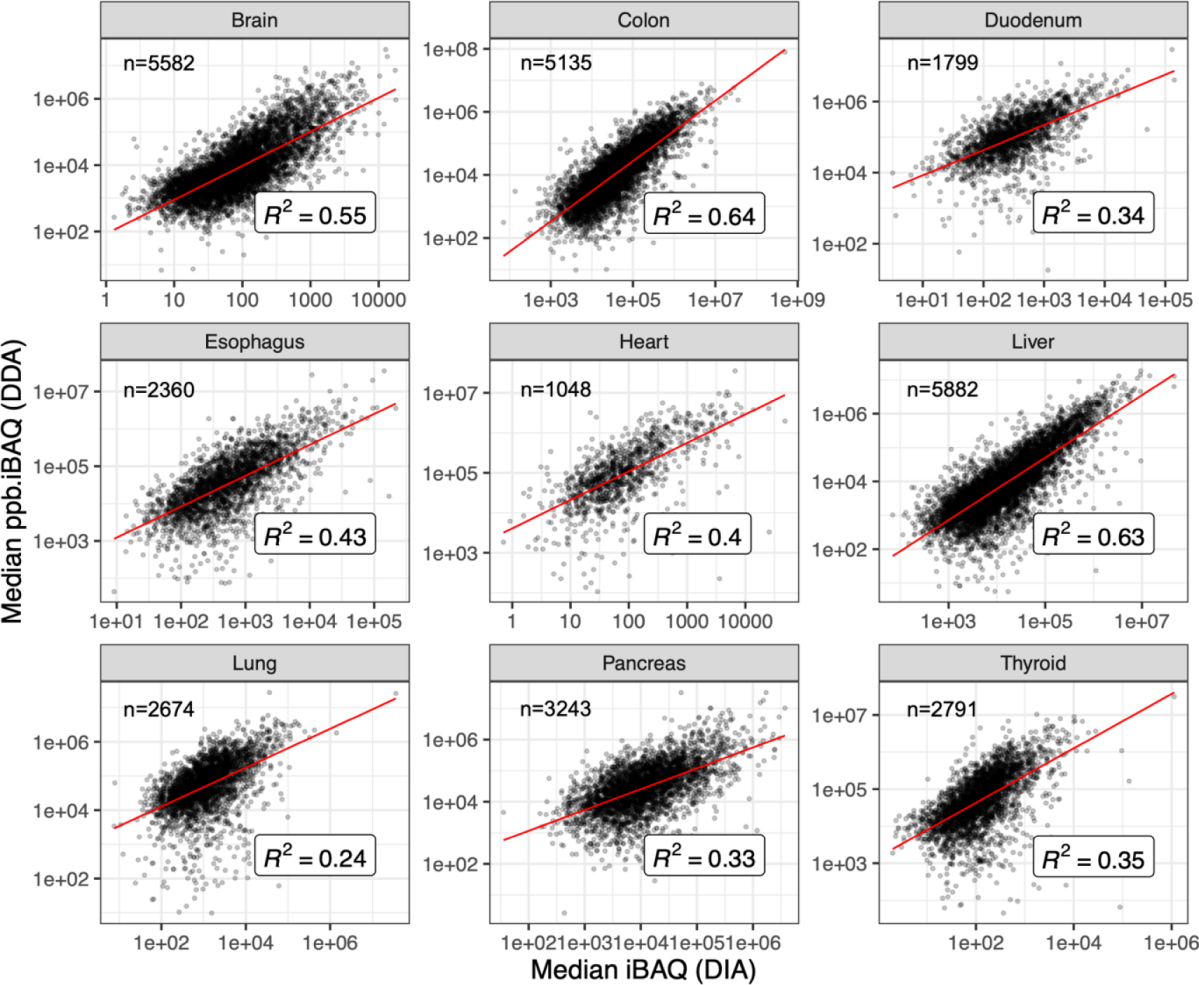
Correlation of protein abundances between human baseline DIA and the DDA data sets from a previous study.^11^*n* shows the number of data points (common canonical proteins) considered in each panel.

We then compared protein abundances between DIA data sets and DDA data sets from ref (11). Protein abundances from DDA data sets were computed as iBAQ values and, as mentioned in “Methods”, the abundances from DIA data sets were transformed from LFQ to iBAQ values. We observed a strong correlation of protein abundances in various tissues, including colon (*R*^2^ = 0.64), liver (*R*^2^ = 0.63), and brain (*R*^2^ = 0.55), and a weak correlation in lung (*R*^2^ = 0.24), pancreas (*R*^2^ = 0.33), duodenum (*R*^2^ = 0.34), and thyroid (*R*^2^ = 0.35) (Figure 5). We also compared the binned protein abundances of the commonly identified proteins between tissues in DIA and DDA data sets^11^ (Supporting Information 3). We observed that highly abundant proteins (of bins 4 and 5) showed a higher similarity in protein abundances between DIA and DDA data sets, when compared to lower abundant proteins (of bins 1 to 3) (Figure S4). The protein abundance profiles in Supporting Information 3 can be useful in identifying proteins with similar or dissimilar abundances between different tissues in DIA and DDA data sets. For example, in Supporting Information 3, it can be observed that the mitochondrial protein ATP synthase group of subunits (gene name: ATP5F1A, UniProt accession: P25705) was detected in both DIA and DDA data sets to be highly expressed across all tissues consistently. However, the γ-aminobutyric acid receptor subunits (gene name: GABRA1, UniProt accession: P14867) were exclusively identified in brain samples from both DIA and DDA data sets.

We then compared our results with protein abundance data from DDA studies available in ProteomicsDB.^18^ We observed a good correlation in protein abundance across various tissues (Figure S5). The strongest correlations were observed in liver (*R*^2^ = 0.71) and colon (*R*^2^ = 0.68) and the lowest in thyroid (*R*^2^ = 0.39). We also compared abundances with protein expression data across various human tissues at baseline conditions from a large-scale study using TMT.^19^ In this case, we did not observe a strong correlation across various tissues (Figure S6).

## Discussion

Here, we present a detailed reanalysis of 15 DIA proteomics data sets from 12 human tissues in baseline conditions. We selected data sets from PRIDE, manually curated them connecting the samples with the raw files, and generated protein abundances for all of them. The objective was 2-fold. First, we wanted to facilitate access to protein abundance data from state-of-the-art DIA proteomics approaches. Second, we wanted to explore their comparability with analogous protein abundance data generated using DDA approaches.

Different benchmarking studies have been published, pointing out the advantages and disadvantages of using different approaches (and software tools) for DIA analysis (e.g., refs^43−45^). High-quality spectral libraries can be generated *in silico* in tools such as DIA-NN including every peptide sequence. However, using a public or a “matched experimental” library, containing peptidoforms likely to be present in the sample (e.g., for particular biological conditions, tissues, or fluids), would likely provide a better statistical power, at the expense of losing a few low-abundant peptidoforms absent from a DDA library. An *in silico* spectral library can be potentially much bigger and thus may give lower sensitivity overall. We here assessed the feasibility of using them to obtain reliable results. It is important to highlight that the use of *in silico* spectral libraries makes public data reanalysis efforts feasible (like in the case of DDA data sets), without having to rely on the availability of, e.g., a “pan-species” public library. The other alternative option is that submitters make their in-house spectral libraries publicly available as well, but at present, this does not happen very often. Indeed, although spectral libraries can be submitted to ProteomeXchange resources (and PRIDE in particular) as part of DIA data sets, it is not mandatory to do it, and then, it is not a common practice yet. However, there are cases where they are made available, and this is a key factor for enabling the reproducibility of the results of the original studies.

The approach followed in this study is different from our previous reanalysis effort of DIA data sets, where the “pan-human” library was used,^17^ but also a different analysis software (the pipeline was based on OpenSWATH).^46^ No common data sets were included in both studies. In the current study, by using an entrapment database, we checked that the resulting protein FDR per data set was at an adequate level.

We also compared the protein abundance values in DIA data sets with the results generated from previous DDA studies. One caveat is that at present, the number of public DIA human data sets from baseline human tissues is much smaller when compared with DDA. The results of this analysis were heterogeneous, with a good level of overall correlation for some of the tissues (especially for liver and colon), but much lower for others (e.g., lung and pancreas). Also, we studied the proteins that were detected by either DDA, DIA, or both approaches and compared their level of expression across the common tissues in either DDA or DIA (see Supporting Information 3). Finally, we observed a similar percentage of protein abundance missing values among samples analyzed by DIA and DDA techniques. It needs to be taken into account that there are some limitations in this comparison. On one hand, samples are not matched between the different studies. On the other hand, for our previous DDA metaanalysis study, we used a different protein sequence database, including several proteins per gene. Lastly, there was only one DIA data set where fractionation was used. The objective of this study was not to identify tissue-specific proteins. Given that most tissues reanalyzed here are only represented by one data set (apart from brain and skin), we think that confidently identifying protein tissue specificity is not feasible (data not shown). However, if the main objective of a study is to find tissue-specific proteins, it would be possible to combine downstream the results found in this study with the findings in our analogous previous DDA study.^11^

Whereas the reuse of public DIA proteomics data sets is relatively common for benchmarking efforts in the context of the development of software tools and analysis approaches (e.g., refs^47−49^) data reanalysis of DIA data sets is still very limited, unlike in the case of DDA data sets. However, the trend is changing as more data make it into the public domain. Some recent efforts such as the development of the open quantMS pipeline^50^ can facilitate the reanalysis of large data sets.

In conclusion, we here present a metaanalysis study of public DIA human data sets generated from tissues in baseline conditions, from the PRIDE database. Analogous studies of protein abundance in model organisms (such as those possible for DDA data) are not feasible yet because the amount of DIA baseline data from them is still small. The resulting protein abundance data have been made available via Expression Atlas.

## Data Availability

All public data sets that have been reanalyzed in this study are listed in Table 1. The scripts used for this work are available at: https://github.com/Ananth-Prakash/PRIDE-DIA-DataReuse.

## Supporting Information Available

Figure S1: comparison of the DIA-NN runtimes of an example data set when run in sequential and parallel modes; Figure S2: Venn diagrams showing the number of canonical proteins identified by DIA in this study and by DDA in our previous study^11^ in various tissue samples; Figure S3: comparison of missing values in samples between DDA^11^ and DIA data sets; Figure S4: comparison of binned protein abundances in tissues from DIA and DDA data sets; Figure S5: comparison of protein expression across various human tissues in baseline conditions analyzed from DIA data sets (this study) and from ProteomicsDB; Figure S6: comparison of protein expression across various human tissues at baseline condition analyzed from DIA data sets (this study) and from Jiang et al.^19^ using the TMT-labeling method (PDF)
Table S1: canonical protein abundances (iBAQ) across various tissues; Table S2: binned canonical protein abundances across various tissues; Table S3: canonical proteins identified by DIA only, DDA only, and by both techniques (XLSX)
Comparison of the protein abundance profiles of all canonical proteins identified between DDA from our previous study^11^ and DIA data sets (PDF)

## Abbreviations

EA: Expression Atlas
DDA: data dependent acquisition
DIA: data independent acquisition
FDR: false discovery rate
iBAQ: intensity-based absolute quantification
IDF: investigation description format
LFQ: label-free quantification
ppb: parts per billion
SDRF: sample and data relationship format
